# COVID‐19 outcomes in haematopoietic cell transplant recipients: A systematic review and meta‐analysis

**DOI:** 10.1002/jha2.465

**Published:** 2022-06-14

**Authors:** Yeong Jer Lim, Umair Khan, Indrani Karpha, Andrew Ross, Muhammad Saif, Mats Remberger, Nagesh Kalakonda, Andrew R. Pettitt, Yngvar Floisand

**Affiliations:** ^1^ Haemato‐oncology Department Clatterbridge Cancer Centre NHS Foundation Trust Liverpool UK; ^2^ Institute of Systems, Molecular and Integrative Biology University of Liverpool Liverpool UK; ^3^ Department of Medical Sciences Uppsala University and KFUE Uppsala University Hospital Uppsala Sweden; ^4^ Centre for Cancer Cell Reprogramming, Institute of Clinical Medicine University of Oslo Oslo Norway

**Keywords:** COVID‐19, SCT, stem cell transplantation

## Abstract

Up‐to‐date information on coronavirus disease 2019 (COVID‐19) outcomes and risk factors in haematopoietic cell transplantation (HCT) recipients is required to inform on decisions about cancer treatment and COVID‐19 mitigation strategies. We performed a meta‐analysis to address this knowledge gap. All studies with at least five patients who reported COVID‐19‐related deaths in HCT recipients were included. The primary outcome was COVID‐19‐related death. Secondary outcomes were COVID‐19‐related mechanical ventilation (MV) and intensive care unit (ITU) admission. The cumulative COVID‐19‐related death rate among HCT recipients was 21% (95% confidence interval [CI] 18%–24%), while MV and ITU admission rates were 14% (95% CI 11%–17%) and 18% (95% CI 14%–22%), respectively. Subgroup analysis showed higher death rates in patients who developed COVID‐19 within 12 months of HCT (risk ratio [RR] 1.82, 95% CI 1.09–3.03), within 6 months of receiving immunosuppressant drugs (RR 2.11, 95% CI 1.38–3.20) or in the context of active graft‐versus‐host disease (RR 2.38, 95% CI 1.10–5.16). Our findings support the idea that HCT should remain an integral part of cancer treatment during the COVID‐19 pandemic but also highlight the need to prioritise preventative measures in those patients who are at increased risk of adverse COVID‐19 outcomes.

## 1 INTRODUCTION

It has been demonstrated that patients with cancer appear particularly vulnerable to coronavirus disease 2019 (COVID‐19) caused by the SARS‐CoV‐2 virus owing in part to the immunosuppressive effects of anticancer therapies [1, 2, 3]. Within this group, haematopoietic cell transplant (HCT) recipients remain one of the most immunosuppressed cohorts and are considered to be at a high risk of COVID‐19‐related complications and death. Despite this, COVID‐19 outcomes for HCT recipients in single‐centre [4, 5, 6] or multicentre studies [7, 8] vary, with different studies reporting different mortality rates and risk factors. Consequently, the true risk of COVID‐19 in HCT recipients is unclear. Addressing this knowledge gap is essential in order to weigh the risks and benefits of HCT during the COVID‐19 pandemic, particularly as recent studies have shown that HCT recipients produce a less effective immune response to COVID‐19 vaccines compared to the general population [9, 10, 11]. In light of these considerations, we performed a systematic review and meta‐analysis to quantify the clinical outcomes of HCT recipients with COVID‐19, as well as a comprehensive subgroup analysis to determine clinical factors associated with adverse outcomes.

## 2 METHODS

This systematic review was performed in accordance with the Preferred Reporting Items for Systematic Reviews and Meta‐Analyses (PRISMA) [12] and the Joanna Briggs Institute (JBI) guidelines.

### 2.1 Inclusion criteria

All studies reporting COVID‐19 outcomes in HCT recipients were screened, and all those reporting COVID‐19‐related deaths in cohorts of at least five patients were selected irrespective of whether they involved paediatric (age <18 years old) or adult (age ≥18 years old) patients.

### 2.2 Search strategy

We identified relevant studies using the PubMed and Embase databases. We also sought preprint articles from medRxiv and bioRxiv, as well as conference proceedings from relevant international scientific meetings organised by the American Society of Clinical Oncology, American Society of Hematology, European Society for Blood and Marrow Transplantation (EBMT) and European Haematology Association. We used the search terms ‘([coronavirus] OR [COVID‐19] OR [SARS‐CoV‐2] OR [COVID‐2019])’ AND ‘([stem cell transplantation] OR [bone marrow transplantation] OR [hematopoietic cell transplantation] OR [HCT] OR [SCT] OR [HCT] OR [BMT])’. This was last updated on 20 December 2021. In addition, reference lists of studies, systematic reviews, narrative reviews and case reports were also scrutinised for eligible studies.

### 2.3 Study selection

Two reviewers (Yeong Jer Lim and Umair Khan) independently evaluated all titles and abstracts identified through the initial search and excluded studies that clearly did not meet the inclusion criteria. The full texts of the remaining studies were evaluated for eligibility, and any differences of opinion were resolved by discussion.

### 2.4 Data extraction

Using a preformulated template, two reviewers (Yeong Jer Lim and Umair Khan) independently extracted the following data: study characteristics (author, centre(s), region, inclusion period), sample size, patient characteristics (age, gender, major comorbidities/haematopoietic cell transplantation‐specific comorbidity index [HCT‐CI] score, indication for HCT), description of HCT received (allogeneic or autologous HCT, conditioning regimen, donor and graft type, graft‐versus‐host disease [GvHD] prophylaxis received, time since transplant, active GvHD or immunosuppressant use at time of COVID‐19), diagnostic criteria for COVID‐19 and duration of follow‐up.

The primary outcome was the cumulative rate of COVID‐19‐related deaths among HCT recipients, defined as death from any cause following a diagnosis of COVID‐19. The secondary outcomes were COVID‐19‐related mechanical ventilation (MV) and intensive care unit (ITU) admission rates, defined as any episode of MV or ITU admission following COVID‐19. Predetermined subgroup analyses were also conducted that related the primary outcome to age, gender, types of HCT, time since HCT, recent immunosuppressant use and active GvHD at the time of COVID‐19 onset.

### 2.5 Risk of bias assessment

All included studies were independently assessed for risk of bias by two reviewers (Yeong Jer Lim and Umair Khan) using the JBI critical appraisal checklist for studies reporting prevalence data [13] (Table S1). Briefly, this checklist comprised nine yes/no answers and assessed the following areas in each study: the method of study participant selection and the suitability of included participants to represent the target population; the adequacy of the sample size reported on; the description of study subjects and setting; the coverage of subgroups of interest; the validity of the identification and assessment of the condition; the suitability of the statistical analysis performed; and the adequacy of the study response rate. The adequacy of the sample size in each study was compared to sample size calculations [14] using an estimated risk of COVID‐19‐related death of 25% with a precision of 0.1 (95% confidence interval [CI] width of 20%). The estimated risk of COVID‐19‐related death was the average cumulative death rate among the two largest available datasets [7, 8] on COVID‐19‐related deaths in HCT patients at the time of writing.

The risk of bias was assessed based on the number of ‘yes’ answers to each question in the checklist. Any disagreements between reviewers were resolved by discussion. Risk of bias was classified as high in studies with one to four out of nine ‘yes’ answers, moderate in studies with five to six ‘yes’ answers and low in studies with seven or more ‘yes’ answers. Furthermore, publication bias was assessed by Begg's funnel plot analysis with Egger's test, and a *p*‐value <0.05 was deemed significant.

### 2.6 Statistical analysis

We used RevMan version 5.3 and the R package ‘meta’ to perform the meta‐analysis and Microsoft Excel 2010 for data compilation. For the primary and secondary outcomes, all included studies were pooled into a single arm forest plot where the combined incidence rate and its 95% CIs were calculated. For subgroup analysis, forest plots were constructed for each predefined subgroup, and a pooled relative risk with 95% CIs was calculated as well as the overall effect of each variable on COVID‐19‐related death rates. The threshold for statistical significance of the overall effect was *p* ≤0.05. Heterogeneity was judged to be significant if *p* ≤0.10 on the chi‐square test, while the assigned *I* ^2^ values of 25%–49.9%, 50%–74.9% and 75%–100% were deemed low, moderate and high degrees of heterogeneity, respectively. A random effects model was used for all statistical analyses. Finally, we have also performed a sensitivity analysis of the primary outcome by excluding studies deemed to have a moderate to high risk of bias, lower sample size (*n* < 10) or those only reporting on paediatric HCT recipients (age <18 years old).

## 3 RESULTS

### 3.1 Study selection

**FIGURE 1:**
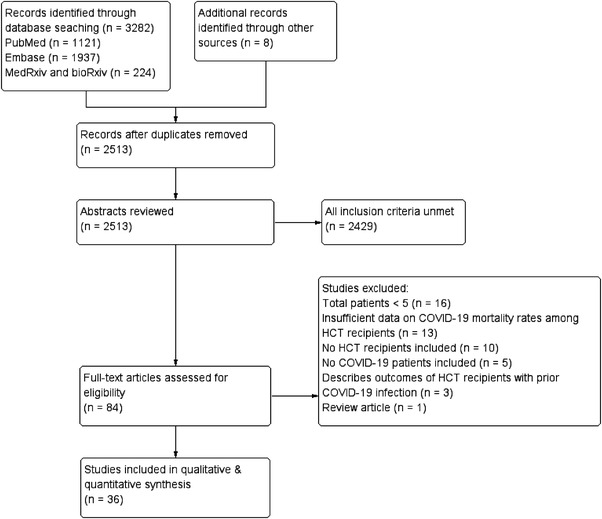
Preferred Reporting Items for Systematic Reviews and Meta‐Analyes (PRISMA) flow diagram detailing how studies were identified and selected

The results of the literature search are summarised in Figure 1. Through a systematic search strategy, we identified 1634 records, of which 94 records were assessed in full text for eligibility. In total, we found 36 records reporting 2141 patients, all in English and published between January 2020 and December 2021, which met the predetermined inclusion criteria. Conversely, 58 records were excluded because they did not meet the inclusion criteria.

### 3.2 Characteristics of the selected studies

Patient demographics and study characteristics are summarised in Table S1. Selected studies reported retrospective observational data from single‐centre experiences [4–6, 15, 16, 17, 18, 19, 20, 21, 22, 23, 24, 25, 26], multicentre experiences [27, 28, 29, 30, 31, 32, 33, 34, 35, 36, 37, 38, 39, 40, 41] or registry data [7, 8, 42, 43, 44, 45]. Seventeen studies [8, 15, 18, 19, 23, 24, 29, 31, 33, 34, 35, 37, 38, 39, 41, 44, 45] were from Europe (1153 patients), seven studies [4, 6, 17, 20, 22, 25, 36] were from North America (238 patients), seven studies [5, 16, 21, 26, 30, 32, 42] were from the Middle East (185 patients), two studies [28, 40] were from South America (41 patients), one study [27] was from Asia (28 patients) and two studies [7, 43] included patients from multiple regions (496 patients). Seventeen studies [7, 8, 16, 21, 24, 25, 26, 27, 28, 29, 30, 35, 38, 39, 40, 41, 45] included both adult and paediatric transplant recipients, 13 studies [4, 5, 6, 15, 18, 19, 20, 22, 23, 34, 36, 42, 43] only included adult patients ≥18 years old and five studies [17, 31, 32, 33, 37, 44] only included patients <18 years old. In most studies [4, 5, 8, 16, 17, 18, 19, 20, 21, 23, 24, 25, 27, 28, 30, 31, 33, 34, 35, 36, 37, 38, 39, 40, 41, 42, 43, 44], the diagnosis of COVID‐19 was based on SARS‐CoV‐2 polymerase chain reaction or SARS‐CoV‐2 IgG antibody (IgG) positivity, while six studies [6, 7, 15, 22, 29, 32] additionally included patients with a strong clinical or radiological suspicion of COVID‐19. Eight studies included outcomes of HCT recipients within larger cohorts of patients with haematological malignancies (HM) [15, 17, 22, 31, 32, 41, 43] or solid organ transplant recipients [29], with limited available information on patient characteristics and treatment.

### 3.3 Characteristics of HCT recipients in the selected studies

Among the 2141 patients included in this meta‐analysis, 944 (44.1%) received an autologous HCT and 1191 (55.6%) received an allogeneic HCT, while details of the transplant were not available for six patients (0.3%). COVID‐19 was acquired within 12 months of HCT in 282 of 863 patients where this information was available (32.7%) and more than 1 year after HCT in 581/863 (67.3%). Within the allogeneic HCT cohort, 247/554 patients (44.6%) received immunosuppression therapy within 6 months of COVID‐19 diagnosis, 185/600 patients (30.8%) had acute GvHD at the onset of COVID‐19, and 194/580 patients (33.4%) had chronic GvHD at the onset of COVID‐19. Further details of allogeneic HCT recipients are summarised in Table S2.

### 3.4 Risk of bias assessment

Using the JBI checklist for prevalence studies, we identified 25 (69%) studies with a low risk of bias and 11 (31%) studies with moderate to high risk. The results for the risk of bias assessment and its justification are summarised in Table 1. When assessing for publication bias, asymmetry was seen in the funnel plot (Figure S1), with Egger's test (*p* = 0.001) showing the presence of significant publication bias in the included studies.

**TABLE 1:** Risk of bias assessments for all selected studies using the Joanna Briggs Institute (JBI) critical appraisal checklist for studies reporting prevalence data

Study	Q1: Was the sample frame appropriate to address the target population?	Q2: Were study participants sampled in an appropriate way?	Q3: Was the sample size adequate?	Q4: Were the study subjects and the setting described in detail?	Q5: Was the data analysis conducted with sufficient coverage of the identified sample?	Q6: Were valid methods used for the identification of the condition?	Q7: Was the condition measured in a standard, reliable way for all participants?	Q8: Was there appropriate statistical analysis?	Q9: Was the response rate adequate, and if not, was the low response rate managed appropriately?	Overall risk of bias
Agrawal et al. 2021	Y	Y	N^*^	Y	Y	Y	Y	Y	Y	Low
Altuntas et al. 2020	Y	Y	N^*^	Y	Y	Y	Y	Y	Y	Low
Averbuch et al. 2021	Y	Y	N^*^	Y	N^∆^	Y	Y	Y	Y	Low
Bailén et al. 2020	Y	Y	Y	Y	Y	U^¥^	U^¥^	Y	Y	Low
Basquiera et al. 2021	Y	Y	N^*^	Y	N^Ω^	Y	Y	Y	Y	Low
Camargo et al. 2021	Y	Y	N^*^	Y	Y	Y	Y	Y	Y	Low
Chari et al. 2020	Y	Y	Y	N^◊^	N^Ψ^, ^γ^	Y	Y	Y	Y	Low
Coll et al. 2020	Y	Y	Y	N^◊^	U^□^	Y	Y	Y	Y	Low
De Ramón et al. 2020	Y	Y	Y	N^◊^	U^□^	Y	Y	Y	Y	Low
Dwabe et al. 2021	Y	Y	N^*^	Y	N^†^	Y	Y	Y	Y	Low
Fakih et al. 2021	Y	Y	Y	Y	Y	Y	Y	Y	Y	Low
Faura et al. 2020	Y	Y	N^*^	N^◊^	N^∆^, ^Ω^	Y	Y	N/A^○^	Y	Mod
Fox et al. 2020	Y	Y	N^*^	N^◊^	N^γ^	Y	Y	Y	Y	Mod
Haroon et al. 2020	Y	Y	N^*^	Y	Y	Y	Y	N/A^○^	Y	Low
Jimenez‐Kurlander et al. 2020	Y	Y	N^*^	N^◊^	N^∆^, ^Ω^	U^θ^	Y	N/A^○^	Y	High
Kanellopoulos et al. 2020	Y	Y	N^*^	Y	Y	Y	Y	N/A^○^	Y	Low
Karatas et al. 2021	Y	Y	N^*^	Y	Y	Y	Y	Y	Y	Low
Kebudi et al. 2020	Y	Y	N^*^	N^◊^	N^∆^	Y	Y	N/A^○^	Y	Mod
Ljungman et al. 2021	Y	Y	Y	Y	Y	Y	Y	Y	Y	Low
Lucchini et al. 2021a	Y	Y	N^*^	Y	N^∆^, ^Ω^	Y	Y	N/A^○^	Y	Mod
Lucchini et al. 2021b	Y	Y	Y	Y	N^Ω^	Y	Y	Y	Y	Low
Lupo‐Stanghellini et al. 2021	Y	Y	N^*^	Y	N^Ω^	Y	Y	N/A^○^	Y	Mod
Machado et al. 2020	Y	Y	N^*^	N^◊^	Y	Y	Y	Y	Y	Low
Malard et al. 2020	Y	Y	N^*^	Y	Y	Y	Y	N/A^○^	Y	Low
Mico et al. 2020	Y	Y	N^*^	Y	N^Ω^	Y	Y	Y	Y	Low
Mushtaq et al. 2021	Y	Y	N^*^	Y	Y	Y	Y	Y	Y	Low
Passamonti et al. 2020	Y	Y	Y	Y	Y	Y	Y	Y	Y	Low
Pinana et al. 2020	Y	Y	Y	Y	Y	Y	Y	Y	Y	Low
Shah et al. 2020	Y	Y	Y	Y	Y	Y	Y	Y	Y	Low
Sharma et al. 2021	Y	Y	Y	Y	Y	Y	Y	Y	Y	Low
Sultan et al. 2020	Y	Y	N^*^	Y	N^∆^, ^Ω^	Y	Y	N/A^○^	Y	Mod
Tailor et al. 2020	Y	Y	N^*^	Y	N^γ^	U^¥^	U^¥^	N/A^○^	Y	Mod
Varma et al. 2020	Y	Y	N^*^	Y	Y	Y	Y	Y	Y	Low
Vicent et al. 2020	Y	Y	N^*^	Y	N^∆^, ^Ω^	Y	Y	N/A^○^	Y	Mod
Wang et al. 2020	Y	Y	N^*^	N^◊^	N^Ψ^, ^γ^	Y	Y	Y	Y	Mod
Xhaard et al. 2021	Y	Y	N^*^	Y	N^Ω^	Y	Y	Y	Y	Low

Abbreviations: HCT, haematopoietic cell transplantation; N, no; N/A, not applicable; SCT, stem cell transplantation; U, unclear; Y, yes.

^*^ Study sample size lower than estimated sample size required using estimated mortality risk of 25%, precision 0.1.

^◊^ Insufficient description of patient/HCT treatment information.

^□^ Insufficient patient/treatment information provided to determine coverage.

^∆^ Only HCT patients <50 years old included.

^○^ Descriptive report no meaningful statistical analysis performed.

^Ω^ Only allogeneic SCT recipients included.

^Ψ^ Only multiple myeloma patients included.

^γ^ Only autologous SCT recipients included.

^θ^ Use of SARS‐CoV‐2 IgG positivity only as the inclusion criteria may introduce selection bias towards survivors of COVID‐19 infection.

^†^ Only one auto‐HCT recipient included.

^¥^ Diagnostic criteria for COVID‐19 infection not provided.

### 3.5 Clinical outcomes

**FIGURE 2:**
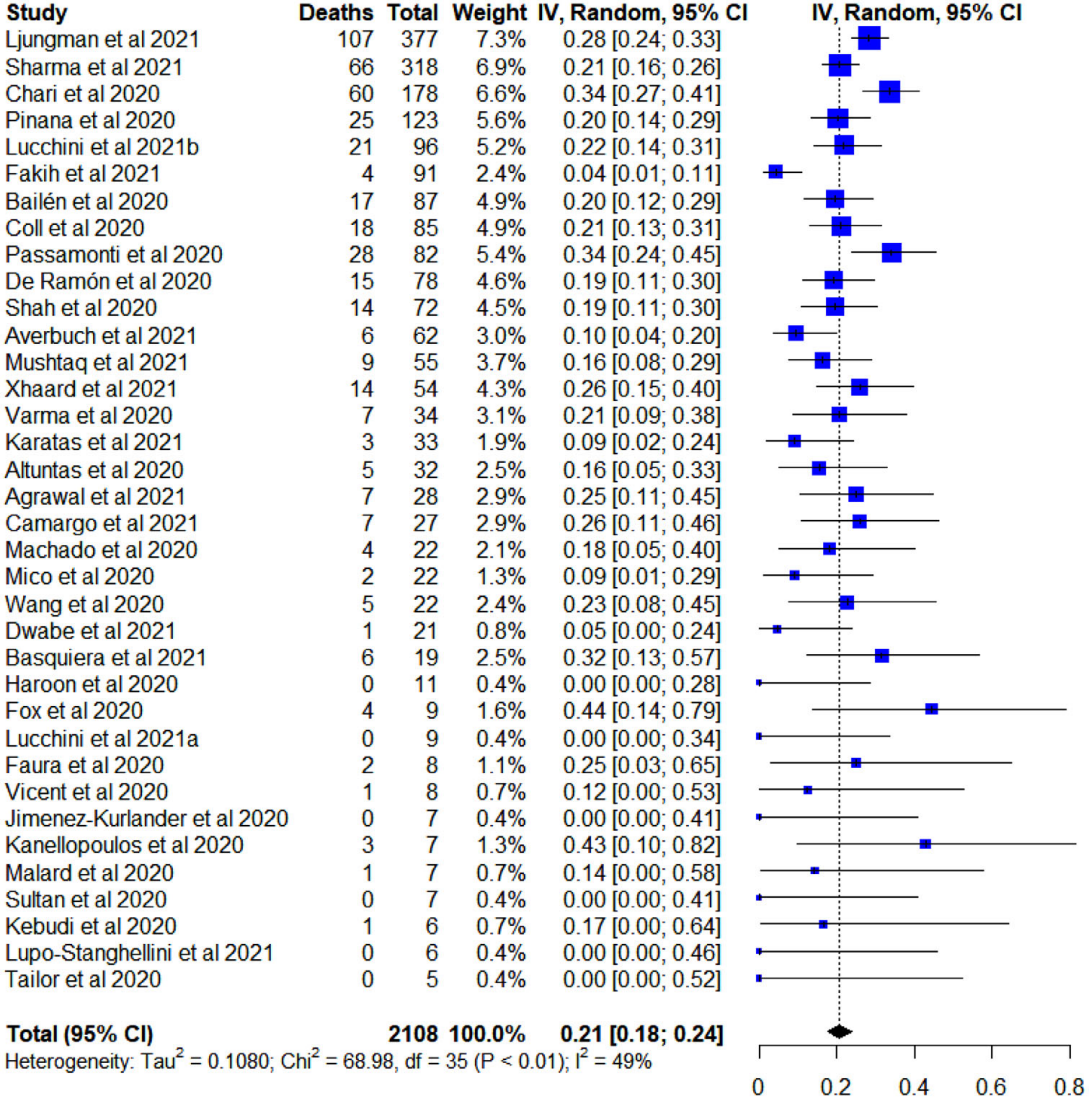
Forest plot of the COVID‐19‐related death rate in all selected studies

Data on COVID‐19 deaths were provided for all patients apart from 33 patients in two studies [8,^29^]. The median follow‐up period ranged from 21 to 282 days. Within the cohort of 2108 informative patients, the overall COVID‐19‐related death rate was 21% (95% CI 18%–24%) (Figure 2). Significant heterogeneity (*I* ^2^ = 49%; χ^2^ = 68.98; df = 35, *p* < 0.01) was detected between different studies.

The overall COVID‐19‐related MV rate was 14% (95% CI 11%–17%) from a total of 868 informative patients, while the ITU admission rate was 18% (95% CI 14%–22%) from a total of 1033 informative patients (Figures S2 and S3). Heterogeneity was found to be statistically significant between studies reporting ITU admission rates (*I* ^2^ = 42%; χ^2^ = 32.90; df = 19, *p* = 0.02) but not between studies reporting COVID‐19‐associated MV rates (*I* ^2^ = 11%; χ^2^ = 21.32; df = 19, *p* = 0.32).

### 3.6 Subgroup analysis

**FIGURE 3:**
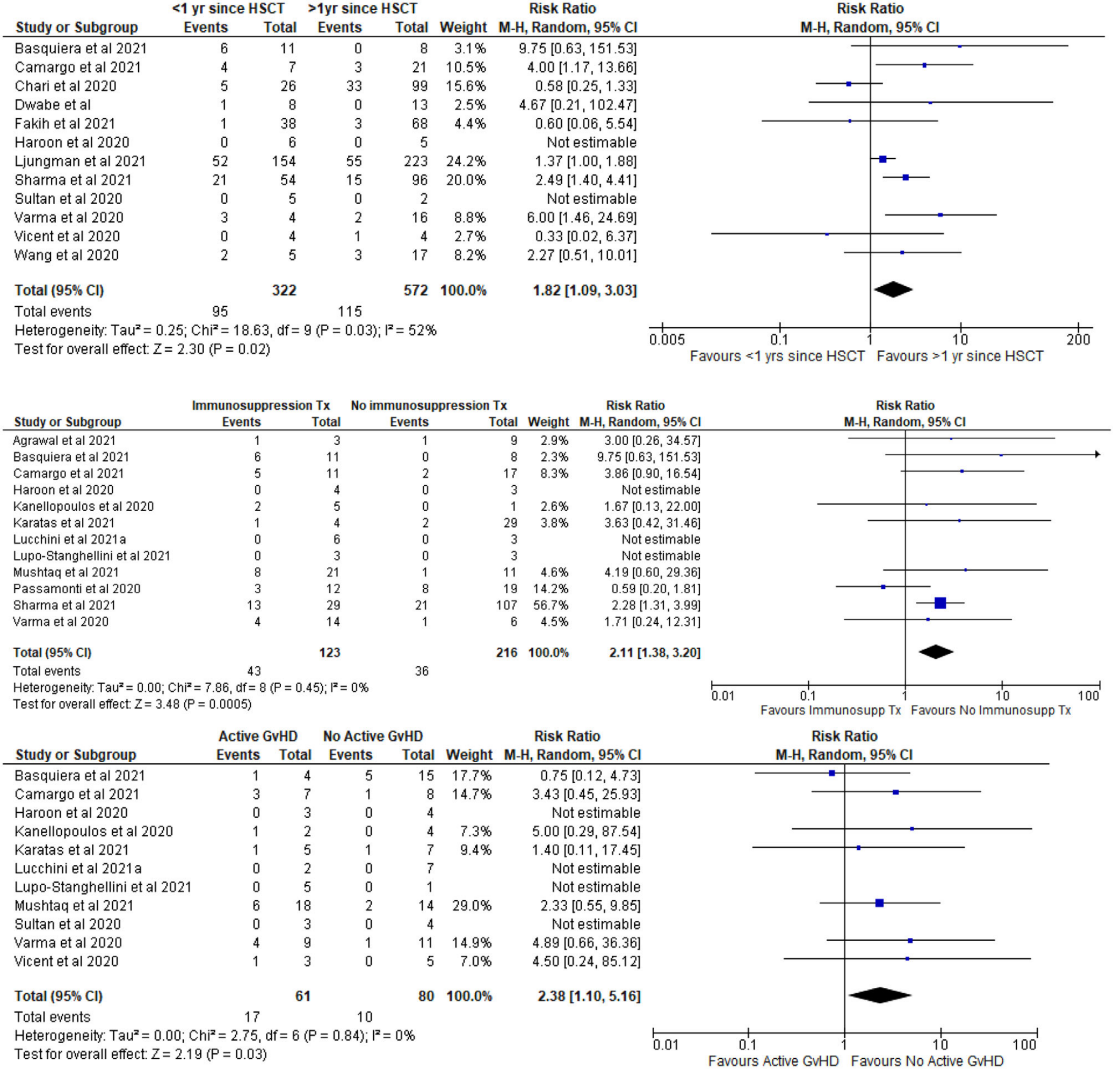
Subgroup analysis of COVID‐19‐related death rate by time from haematopoietic cell transplantation (HCT) to COVID‐19 onset (>1 year vs. <1 year; top), immunosuppressant therapy at the onset of COVID‐19 (middle), and active graft‐versus‐host disease (GvHD) at the onset of COVID‐19 (bottom)

Six predetermined variables were evaluated for their association with the primary outcome. Information on COVID‐19‐related deaths in relation to the time interval between transplant date and COVID‐19 onset was available in 894 patients from 12 studies. The death rate was significantly higher among patients who developed COVID‐19 within 12 months of HCT than among those who had their transplant more than 1 year previously (risk ratio [RR] 1.82, 95% CI 1.09–3.03; *p* = 0.02) (Figure 3). Significant heterogeneity was found between the 11 studies (*I* ^2^ = 52%; χ^2^ = 18.63; df = 9, *p* = 0.03).

Information on COVID‐19‐related deaths in relation to the use of immunosuppressant drug treatment to prevent GvHD was available in 339 patients from 12 studies. The death rate was significantly higher among patients who developed COVID‐19 within 6 months of receiving pharmacological immunosuppression than among those patients whose immunosuppression had been discontinued more than 6 months before the onset of COVID‐19 (RR 2.11, 95% CI 1.38–3.18; *p* = 0.0005) (Figure 3). No significant heterogeneity was found among the 12 studies (*I* ^2^ = 0%; χ^2^ = 7.86; df = 8, *p* = 0.45).

Information on COVID‐19‐related deaths in relation to active GvHD was available in 141 patients from 11 studies. The death rate was significantly higher among patients with active GvHD at the time of COVID‐19 onset than among those with no active GvHD (RR 2.38, 95% CI 1.10–5.16; *p* = 0.03) (Figure 3). No significant heterogeneity was found among the 11 studies (*I* ^2^ = 0%; χ^2^ = 2.75; df = 6, *p* = 0.84).

Notably, we did not observe any significant differences in the COVID‐19‐related death rate when comparing HCT recipients by age (<50 years vs. ≥50 years old; 208 patients from six studies), sex (697 patients from 12 studies) or type of transplant (autologous vs. allogeneic HCT; 1769 patients from 19 studies) (Figure S4).

### 3.7 Sensitivity analysis

Sensitivity analysis showed that the cumulative COVID‐19‐related death rate was not significantly affected by excluding studies with moderate or high risk of bias or with a low sample size (*n* < 10) or by excluding paediatric (age ≤18 years old) HCT recipients or those without laboratory confirmation of SARS‐CoV‐2 infection (Figure S5).

## 4 DISCUSSION

To our knowledge, this meta‐analysis, which utilised published data involving 2141 patients from 36 studies in four different continents, is the first to examine COVID‐19 outcomes in HCT recipients specifically. The cumulative COVID‐19‐related death rate was 21% (95% CI 18%–24%), with MV and ITU admission rates of 14% (95% CI 11%–17%) and 18% (95% CI 14%–22%), respectively. A significantly higher rate of COVID‐19‐related deaths was observed among HCT recipients who developed COVID‐19 within 1 year of HCT (RR 1.82, 95% CI 1.09–3.03; *p* = 0.02), within 6 months of receiving immunosuppressant drugs (RR 2.11, 95% CI 1.38–3.18, *p* = 0.0005), or in the setting of active GvHD (RR 2.38, 95% CI 1.10–5.16; *p* = 0.03). Crucially, some of these associations were not observed in individual studies and became apparent only when the data were pooled. The validity of our findings is supported by the sensitivity analysis, which demonstrated that the COVID‐19 death rate remained unchanged despite excluding studies with a moderate or high risk of selection bias or studies with a lower sample size or by excluding paediatric (age ≤18 years old) HCT recipients or those without laboratory confirmation of SARS‐CoV‐2 infection.

Interestingly, we found no statistically significant difference in the rate of COVID‐19‐related death between males versus females, allogeneic versus autologous HCT recipients, or HCT recipients aged ≥50 years versus <50 years. We believe the latter observation should be interpreted with caution for two reasons. First, due to inconsistency in reporting patient age groups, we were only able to obtain this information in 208 patients from six studies, which represents only 9.7% of the HCT recipients included in this review. Second, since age is a continuous variable, the risk of COVID‐19‐related death may vary significantly depending on which age cutoff is used. We chose a value of 50 years, as it was close to the median age of HCT recipients in most of our included studies. However, it may not have been optimal in separating patients into two groups based on COVID‐19 outcomes. The comparable COVID‐19‐related death rate between allogeneic and autologous HCT recipients is also surprising given the increased risk of posttransplant‐related complications and the degree of immunosuppression associated with allogeneic HCT. This observation was also supported by the two largest multicentre studies [7,^8^] of COVID‐19 outcomes in HCT recipients to date, which showed no significant difference in the overall survival between allogeneic and autologous HCT recipients at 4–6 weeks following COVID‐19. We suspect that these adverse risk factors associated with allogeneic HCT could be offset by the increased age generally found in autologous HCT recipients within our cohort of patients (where available, the median age among allogeneic HCT recipients ranged between 10 and 64 years old, while in autologous HCT, it was 40–65 years old). Furthermore, the variations in comorbidities and HCT indications between both HCT types may also have contributed to this observation, although we did not have sufficient pooled data to make any meaningful analysis in these two areas.

We did observe significant heterogeneity in the COVID‐19‐related death rate and ITU admission between different studies (χ^2^ = 68.98; *p* < 0.01 and chi^2 ^= 32.90; *p* = 0.02, respectively). This likely reflects variation in the proportion of patients with risk factors for adverse COVID‐19 outcomes, including but not confined to the three identified in our subgroup analysis.

We recognise several limitations to our study. First, although our pooled subgroup analysis showed a significantly higher risk of COVID‐19‐related deaths in the first year following HCT, it is difficult to ascertain where these deaths were caused by the SARS‐CoV‐2 virus itself or by pre‐existing or subsequently acquired posttransplant‐related complications unrelated to the diagnosis of COVID‐19, which are known to be higher in the first year posttransplant [46]. Additionally, in most centres HCT recipients are followed up more closely within the first year. This may introduce reporting bias within this cohort of patients compared to those transplanted more than a year ago receiving less intensive follow‐up. We suspect that these two factors may have contributed to the significant heterogeneity seen within this subgroup analysis (χ^2^ = 18.63; *p* < 0.03).

Second, although a higher rate of COVID‐19‐related deaths was observed among HCT recipients in the setting of active GvHD, the information available for this subgroup of HCT recipients was insufficiently detailed to make any meaningful observation on whether this reflects those with acute versus chronic GvHD.

Third, we found evidence of duplicate publication bias. For example, we identified one study [7] involving Center for International Blood and Marrow Transplant Research data where some patient data may have been published separately as single‐centre experiences [6, 35], while another study [8] utilising EBMT registry data may have included data that were reported separately in other studies. Such ‘double reporting’ could potentially amplify the contribution of certain cases in determining the characteristics of the overall study population and therefore distort its true profile. In addition, limitations in resources and COVID‐19 testing capability during the initial stages of the pandemic are likely to have introduced selection bias towards symptomatic or hospitalised patients.

Fourth, due to variation in reporting across the selected studies, insufficient pooled data were available to meaningfully assess potential risk factors for adverse COVID‐19 outcomes such as comorbidity and performance status. However, we did identify five studies [7, 8, 28, 30, 38] that reported comorbidities and/or baseline fitness levels of HCT recipients and their correlation with COVID‐19‐related death rates. Despite consistencies in methods used to assess these risk factors, the presence of comorbidities [28, 30, 38] or variation in baseline fitness (assessed by ECOG performance status [8] or HCT‐CI score [7]) did not appear to correlate with a higher COVID‐19‐related death rate in any of these studies. Variation in reporting also precluded the meaningful analysis of other potential risk factors, including HCT conditioning regimen, indication for HCT, donor type or GvHD prophylaxis regimen used, as well as the effectiveness of different antiviral or anti‐inflammatory therapeutic interventions.

Finally, since all contributing studies were performed prior to COVID‐19 vaccine roll‐out, our meta‐analysis cannot provide direct information on COVID‐19 outcomes and risk factors in vaccinated HCT recipients. However, recent studies [9–11] have reported reduced immunogenicity of COVID‐19 vaccines among HCT recipients compared to the general population. This suggests that the COVID‐19 death rate and risk factors identified in our meta‐analysis may not be substantially different in the postvaccination era.

The COVID‐19 outcomes observed in our meta‐analysis of HCT recipients are notably better than those reported in a recent meta‐analysis [3] of unselected patients with HM who acquired COVID‐19 over the same time period. The latter study showed a pooled COVID‐19‐related death rate of 34% (95% CI 28%–39%) with MV rates and ITU admission rates of 17% and 21%, respectively. The differences in COVID‐19 outcomes between this study and ours may be explained by the stringent patient selection process for HCT, which is likely to result in enrichment of features such as minimal comorbidity, good performance status and control of the underlying disease. In keeping with this idea, large studies reporting on COVID‐19 outcomes in solid organ transplant recipients—another cohort of patients who undergo stringent selection—reported COVID‐19‐related death rates ranging from 19% to 27% [29, 47, 48, 49, 50]. In fact, the COVID‐19‐related death rate observed in our study of HCT recipients is similar to that of unselected patients hospitalised with COVID‐19 during the same time period (February–April 2020) [51, 52].

In summary, this meta‐analysis describes COVID‐19 outcomes in 36 studies involving 2141 HCT recipients across the globe. We found the COVID‐19‐related death rate to be 21%—lower than that of unselected patients with HM and similar to that of patients hospitalised with COVID‐19 in the general population during the same time period. COVID‐19‐related deaths were increased among HCT recipients who developed COVID‐19 within 1 year of HCT, within 6 months of receiving treatment with immunosuppressants, or in the context of active GvHD. These novel observations support the idea that HCT should remain an integral part of HM treatment protocols during the COVID‐19 pandemic. However, HCT recipients who are at increased risk of COVID‐19‐related death should be prioritised for surveillance and preventative measures.

## FUNDING INFORMATION

No funding was received for this meta‐analysis.

## AUTHOR CONTRIBUTIONS

Yeong Jer Lim, Yngvar Floisand and Muhammad Saif conceptualised the study. Yeong Jer Lim and Umair Khan performed the literature search, study selection and data analysis. Yeong Jer Lim wrote the first draft of the manuscript. Andrew R. Pettitt, Nagesh Kalakonda, Mats Remberger, Andrew Ross, Indrani Karpha, Muhammad Saif and Yngvar Floisand contributed to the subsequent edits of the manuscript.

## CONFLICTS OF INTEREST

Andrew Ross, Indrani Karpha, Muhammad Saif and Yngvar Floisand are employed by Clatterbridge Cancer Centre NHS Foundation Trust. Yeong Jer Lim, Andrew R. Pettitt and Nagesh Kalakonda are employed by the University of Liverpool. Yeong Jer Lim is supported by the NW MRC fellowship scheme (award number MR/N025989/1). Mats Remberger is employed by Uppsala University Hospital, Sweden and supported by grants from the Swedish Research Council (VR 2017‐00355).

## PATIENT CONSENT STATEMENT

Not applicable.

## Supporting information

Supporting Information

Supporting Information

## Data Availability

The extracted data are available upon request from the corresponding author.
