# Supplementary material for: COVID‐19 outcomes in haematopoietic cell transplant recipients: A systematic review and meta‐analysis
Source: EJHaem. 2022 Jun 14;3(3):862–72. doi: 10.1002/jha2.465 (PMC9350043; doi:10.1002/jha2.465)
Supplement: Supplementary file 1 — Supporting Information [file JHA2-3-862-s002.docx]

Search Terms:

| PubMed (Date Run: 30/12/2021) | | |
| --- | --- | --- |
| Step | Search term(s) | Results |
| #1 | ((coronavirus) OR (COVID-19) OR (SARS-CoV-2) OR (COVID-2019)) AND ((stem cell transplantation) OR (bone marrow transplantation) OR (hematopoietic cell transplantation) OR (HCT) OR (SCT) OR (HCT) OR (BMT)) | 1121 |

| Embase (Date Run: 30/12/2021) | | |
| --- | --- | --- |
| Step | Search term(s) | Results |
| #1 | 'coronavirus' OR 'covid 19' OR 'sars cov 2' OR 'covid 2019' | 240533 |
| #2 | 'stem cell transplantation' OR 'bone marrow transplantation' OR 'hematopoietic cell transplantation' OR sct OR hct OR bmt | 319637 |
| #3 | #1 AND #2 | 1937 |

| MedRxiv and bioRxiv (Date Run: 30/12/2021) | | |
| --- | --- | --- |
| Step | Search term(s) | Results |
| #1 | ((coronavirus) OR (COVID-19) OR (SARS-CoV-2) OR (COVID-2019)) AND (stem cell transplantation) | 183 |
| #2 | ((coronavirus) OR (COVID-19) OR (SARS-CoV-2) OR (COVID-2019)) AND (bone marrow transplantation) | 31 |
| #3 | ((coronavirus) OR (COVID-19) OR (SARS-CoV-2) OR (COVID-2019)) AND (hematopoietic cell transplantation) | 10 |

| Manual search from conference abstracts from American Society of Clinical Oncology (ASCO), American Society of Hematology (ASH), European Society for Blood and Marrow Transplantation (EBMT) and European Haematology Association (EHA) (Date Run: 30/12/2021) | |
| --- | --- |
| Total eligible abstracts found: | 8 |

Studies excluded:

| Reason | Study author/year: | Total: |
| --- | --- | --- |
| Total patients < 5 | Lu et al 2020, Niu et al 2020, Hatzl et al 2020, Dhakal et al 2020, Huang et al 2020, Balashov et al 2020, Zamperlini-Netto et al 2020, Saraceni et al 2020, Zhou et al 2020, Onaka et al 2020, Cristanziano et al 2020, Sarbay et al 2020, Malek et al 2020, Rossoff et al 2020, Nazon et al 2020, Alexandra et al 2021. | 16 |
| Insufficient data on COVID-19 mortality rates among HCT recipients | Madhusoodhan et al 2020, Shah et al 2020, Rouger-Gaudichon et al 2020, Tanhehco et al 2020, Oshima et al 2020, Mukkada et al 2021, van Doesum et al 2020, Andre et al 2020, Al Yazidi et al 2020, Bisogno et al 2020, Lázaro del Campo et al 2020, Vasquez et al 2020, Garnica et al 2020. | 13 |
| No HCT recipients included | Kalicińska et al 2021, Zhang et al 2020, Passamonti et al 2020, He et al 2020, Yigenoglu et al 2020, Millen et al 2021, Mousavi et al 2020, Robilotti et al 2020, Ghandili et al, 2020, Roedl et al 2021. | 10 |
| No COVID-19 patients included | Valentini et al 2020, Maurer et al 2020, Christopeit et al 2021, Amicucci et al 2020, Rivera-Franco et al 2020. | 5 |
| Describes outcomes of HCT recipients with prior COVID-19 infection | Stanley et al 2021, Jarmoliński et al 2020, Gupta et al 2020. | 3 |
| Review article | Devine 2020 | 1 |

| Question | Responses: | | | |
| --- | --- | --- | --- | --- |
|  | Yes | No | Unclear | Not applicable |
| 1. Was the sample frame appropriate to address the target population? |  |  |  |  |
| 1. Were study participants sampled in an appropriate way? |  |  |  |  |
| 1. Was the sample size adequate? |  |  |  |  |
| 1. Were the study subjects and the setting described in detail? |  |  |  |  |
| 1. Was the data analysis conducted with sufficient coverage of the identified sample? |  |  |  |  |
| 1. Were valid methods used for the identification of the condition? |  |  |  |  |
| 1. Was the condition measured in a standard, reliable way for all participants? |  |  |  |  |
| 1. Was appropriate statistical analysis used? |  |  |  |  |
| 1. Was the response rate adequate, and if not, was the low response rate managed appropriately? |  |  |  |  |

Table S1: JBI Critical Appraisal Checklist for Studies Reporting Prevalence Data.


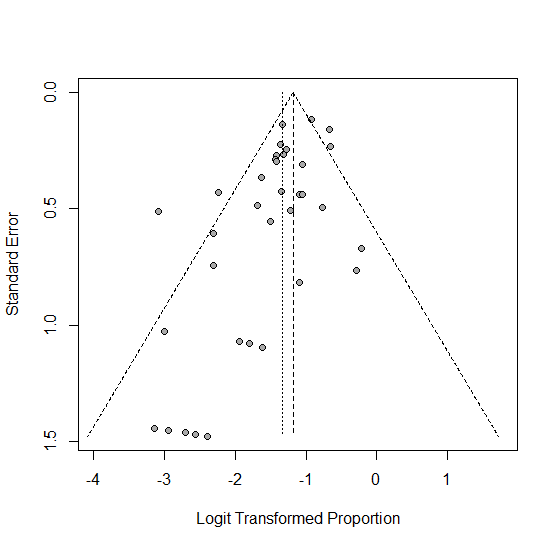


Egger test: t = -3.62, df = 34, P = 0.001

Supplementary figure 1: Funnel plot and egger’s test of effect sizes for publication bias.


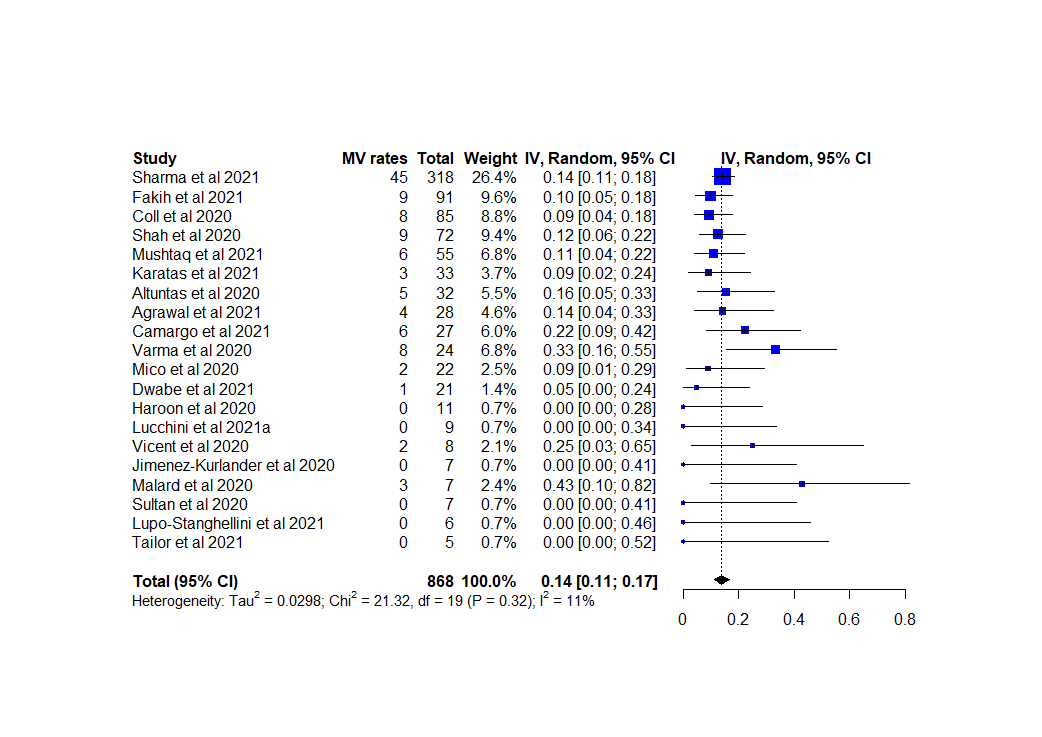


Supplementary figure 2: Forest plot of COVID-19 related MV rates.


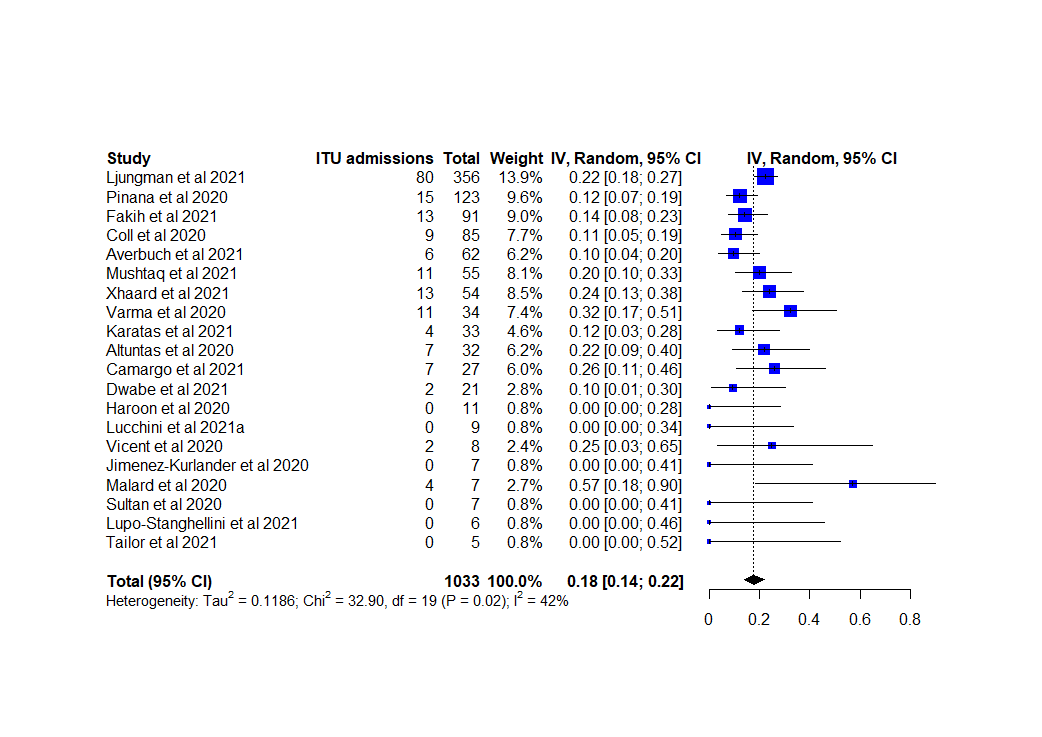


Supplementary figure 3: Forest plot of COVID-19 related ITU admission rates.


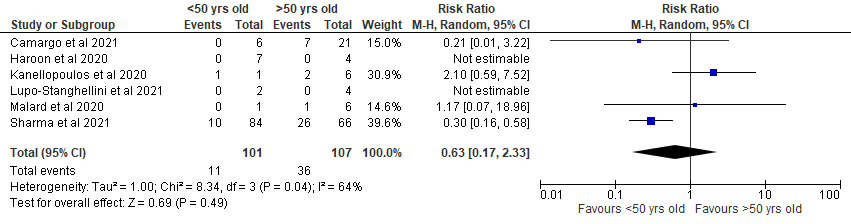


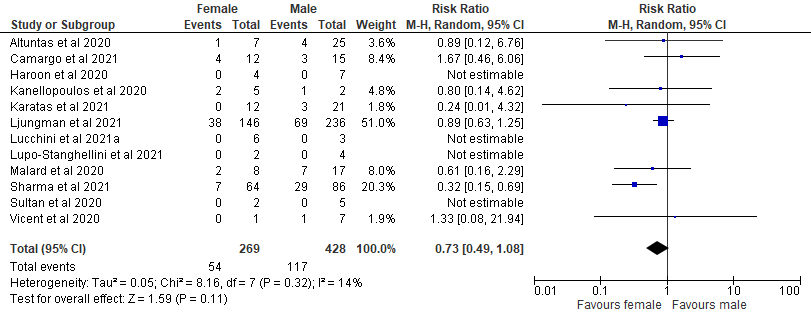


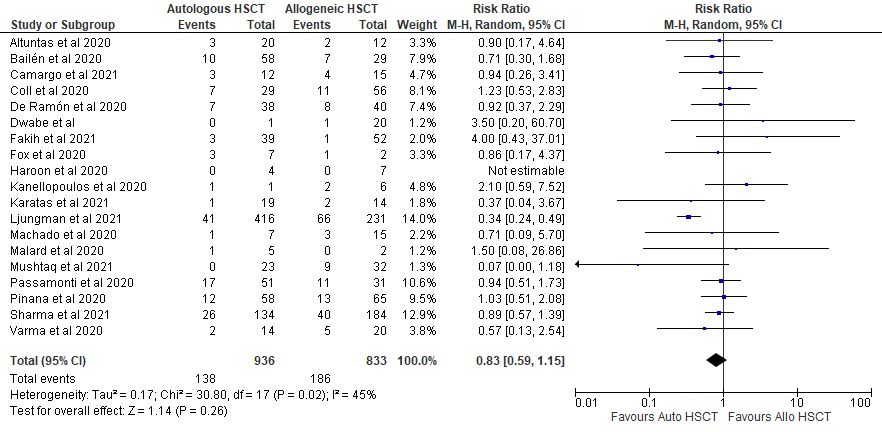


Supplementary figure 4: Subgroup analysis of COVID-19 related death rate by age (<50 vs >50; top), sex (middle) and type of transplant (autologous vs allogeneic; bottom).


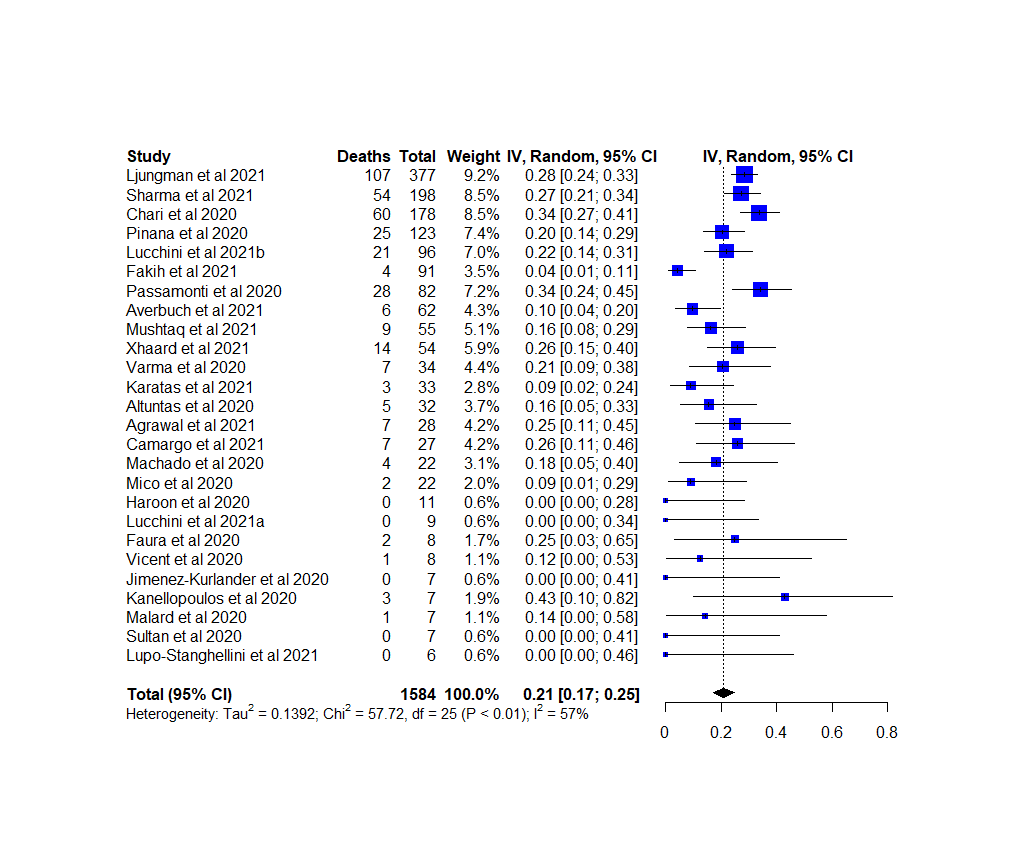

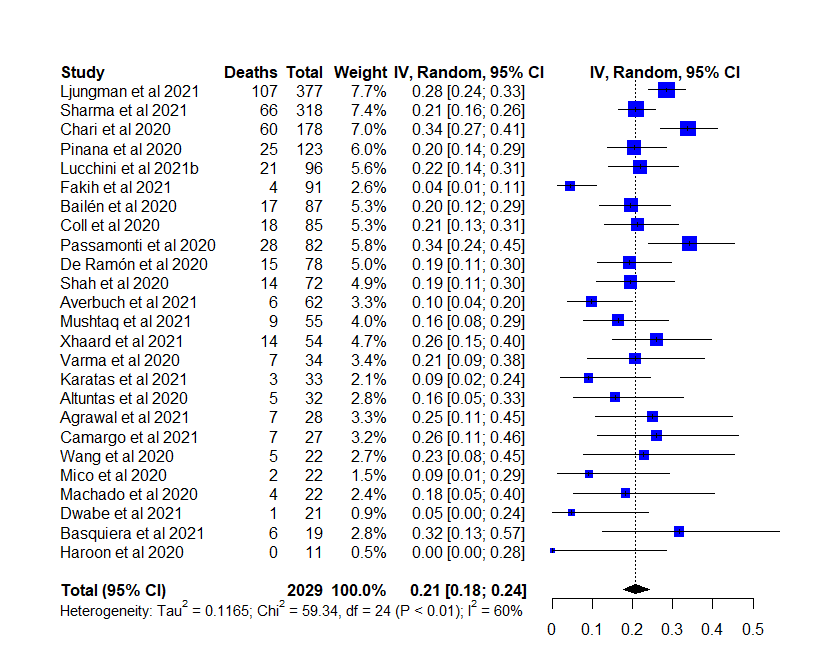

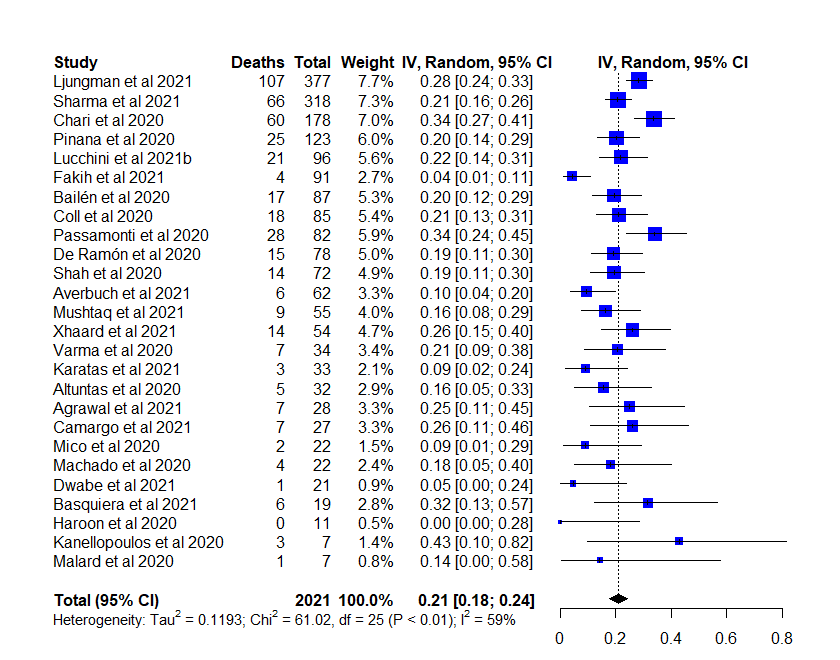


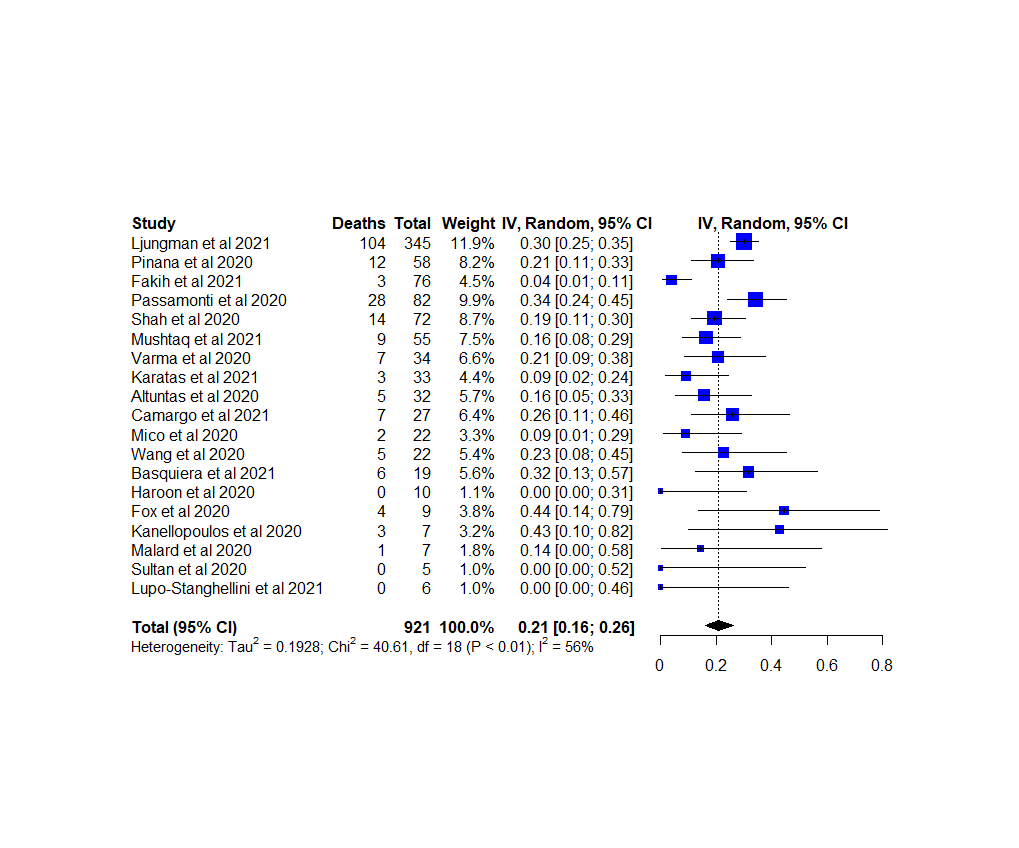


Supplementary figure 5: Sensitivity analysis of COVID-19 related death rate. Forest plots of COVID-19 related death rate excluding studies with moderate to high risk of bias (top left); studies with a low sample size (n <10) (top right); following the exclusion of paediatric HCT recipients (age ≤ 18 years old) (bottom left)*; and following the exclusion of HCT recipients without laboratorial confirmation of SARS-CoV-2 infection (bottom right). *Studies that included outcomes of paediatric HCT recipients without specifying the COVID-19 related death rate among HCT recipients above 18 years old were also excluded.
