# Supplementary material for: COVID‐19 outcomes in haematopoietic cell transplant recipients: A systematic review and meta‐analysis
Source: EJHaem. 2022 Jun 14;3(3):862–72. doi: 10.1002/jha2.465 (PMC9350043; doi:10.1002/jha2.465)
Supplement: Supplementary file 2 — Supporting Information [file JHA2-3-862-s001.docx]

Table 1: Characteristics of selected studies. HCT-CI score: Haematopoietic cell transplantation-specific comorbidity index; HCT: Haematopoietic cell transplantation; MDS/MPN: Myelodysplastic syndrome/myeloproliferative disorders; LPD: lymphoproliferative disorders; PCD: plasma cell dyscrasias; L: laboratory confirmation by SARS-CoV-2 PCR or IgG positivity; C: strong clinical suspicion of COVID-19; R: Radiological suspicion of COVID-19. *3 CAR-T recipients included in patient demographic information. ◊ study included HCT patients retrospectively and prospectively.

Table 2: Characteristics of allogeneic HSCT recipients in the selected studies. MA: myelo-ablative conditioning; RIC: reduced intensity conditioning; MRD: matched related donor; MUD: matched unrelated donor; Mis-UD: mismatched unrelated donor; Haplo: haploidentical donor; BM: bone marrow; PB: peripheral blood; post-tnpt CTX: post-transplant cyclophosphamide; CI: calcineurin inhibitor; MMF: Mycophenolate mofetil; MTx: methotrexate; ATG: Antithymocyte globulin; GvD: graft-versus-host-disease
